# 

**Published:** 1845-10-01

**Authors:** 


BUFFALO

### MEDICAL JOURNAL.

EDITED BY

AUSTIN FLINT, M. D.

**Vol. 1.** **BUFFALO, OCTOBER, 1845.** **No 5.**

### CONTENTS:

ORIGINAL COMMUNICATIONS.

Notes of an European Tour. By F. H. HAMILTON, M. D., Prof. of Surgery in Geneva Medical College. P. 97
Case of Cystotomia; communicated by JAMES P. WHITE, M. D. - - - - - - - - - - 104
Case of fracture of Skull, with loss of Cerebral substance; communicated by ALDEN S. SPRAGUE, M.D. 108
Communication of "ACCOUCHEUR." - - - - - - - - - - - - - - - 110
Case of Morbus Cordis,—Ossification of Sigmoid Valves; communicated by the Editor. - - - - 111

EDITORIAL, MEDICAL INTELLIGENCE, ETC.

Opium, in fever, in large doses. - - - - - - - - - - - - - - - - 113
Delivery of the Placenta before the Child, in cases of Placenta praevia. - - - - - - - - - 114
Medical Fees. - - - - - - - - - - - - - - - - - - - - 115
Medical Schools. - - - - - - - - - - - - - - - - - - - 117
Graduated Wine Glasses. - - - - - - - - - - - - - - - - 119
Meteorological Register. - - - - - - - - - - - - - - - - 119
Obituary. - - - - - - - - - - - - - - - - - - - - 119
Buffalo City Medical Association. - - - - - - - - - - - - - - 119
Physiological effects of Conium Maculatum. - - - - - - - - - - - - 120
National Medical Convention. - - - - - - - - - - - - - - - 120
Notices of New Works, &c. - - - - - - - - - - - - - - See Cover

BUFFALO:
C. F. S. THOMAS, PRINTER AND PUBLISHER,
Exchange Buildings, Third Story, Main-Street.

